# The functional serotonin 1a receptor promoter polymorphism, rs6295, is associated with psychiatric illness and differences in transcription

**DOI:** 10.1038/tp.2015.226

**Published:** 2016-03-01

**Authors:** Z R Donaldson, B le Francois, T L Santos, L M Almli, M Boldrini, F A Champagne, V Arango, J J Mann, C A Stockmeier, H Galfalvy, P R Albert, K J Ressler, R Hen

**Affiliations:** 1 New York State Psychiatric Institute, New York, NY, USA; 2 Department of Psychiatry, Columbia University, New York, NY, USA; 3 Neuroscience, Ottawa Hospital Research Institute, University of Ottawa, Ottawa, ON, Canada; 4 Department of Psychiatry, Emory University, Atlanta, GA, USA; 5 Department of Psychology, Columbia University, New York, NY, USA; 6 Department of Psychiatry and Human Behavior, University of Mississippi Medical Center, Jackson, MS, USA

## Abstract

The G/C single-nucleotide polymorphism in the serotonin 1a receptor promoter, rs6295, has previously been linked with depression, suicide and antidepressant responsiveness. *In vitro* studies suggest that rs6295 may have functional effects on the expression of the serotonin 1a receptor gene (*HTR1A*) through altered binding of a number of transcription factors. To further explore the relationship between rs6295, mental illness and gene expression, we performed dual epidemiological and biological studies. First, we genotyped a cohort of 1412 individuals, randomly split into discovery and replication cohorts, to examine the relationship between rs6295 and five psychiatric outcomes: history of psychiatric hospitalization, history of suicide attempts, history of substance or alcohol abuse, current posttraumatic stress disorder (PTSD), current depression. We found that the rs6295G allele is associated with increased risk for substance abuse, psychiatric hospitalization and suicide attempts. Overall, exposure to either childhood or non-childhood trauma resulted in increased risk for all psychiatric outcomes, but we did not observe a significant interaction between rs6295 and trauma in modulating psychiatric outcomes. In conjunction, we also investigated the potential impact of rs6295 on *HTR1A* expression in postmortem human brain tissue using relative allelic expression assays. We found more mRNA produced from the C versus the G-allele of rs6295 in the prefrontal cortex (PFC), but not in the midbrain of nonpsychiatric control subjects. Further, in the fetal cortex, rs6295C allele exhibited increased relative expression as early as gestational week 18 in humans. Finally, we found that the C:G allelic expression ratio was significantly neutralized in the PFC of subjects with major depressive disorder (MDD) who committed suicide as compared with controls, indicating that normal patterns of transcription may be disrupted in MDD/suicide. These data provide a putative biological mechanism underlying the association between rs6295, trauma and mental illness. Moreover, our results suggest that rs6295 may affect transcription during both gestational development and adulthood in a region-specific manner, acting as a risk factor for psychiatric illness. These findings provide a critical framework for conceptualizing the effects of a common functional genetic variant, trauma exposure and their impact on mental health.

## Introduction

Substantial evidence supports the role of serotonin 1a receptors (5-HT1A) in modulating anxiety and depression-related traits and impacting antidepressant efficacy.^1^ 5-HT1A receptors are highly expressed in areas with strong involvement in emotional and affective processes, including the hippocampus, cingulate cortex, orbitofrontal cortex, insula, amygdala and midbrain raphe.^2, 3^ Positron emission tomography imaging studies have found altered 5-HT1A levels in major depressive disorder (MDD),^4, 5, 6, 7, 8^ bipolar disorder^9^ and anxiety disorders.^10, 11, 12^ Postmortem data also point toward altered 5-HT1A density in depression and suicide.^13, 14, 15, 16, 17, 18^ Clinically, the 5-HT1A partial agonist, buspirone, is used in the treatment of anxiety disorders^19^ and vilazodone, a partial 5-HT1A agonist and serotonin reuptake inhibitor, is used in the treatment of depression.^20^

Work in animal models has also highlighted the distinct behavioral contributions of 5-HT1A receptors across different brain regions and at different developmental time points.^21^ Pharmacological studies have indicated that postnatal but not adult administration of a 5-HT1A antagonist leads to increased anxiety later in life,^22, 23^ and genetic studies suggest that this effect is mediated by 5-HT1A autoreceptors localized on serotonergic neurons in the raphe.^24, 25^ In contrast, manipulations of 5-HT1A in the raphe selectively during adulthood has no effects on anxiety, but does impact stress-coping behaviors and antidepressant responsiveness.^26^ In the forebrain, whole-life suppression of 5-HT1A levels may affect depression-related behavior.^27^ Thus, the forebrain and midbrain 5-HT1A populations modulate distinct behaviors, and their contribution to behavior evolves across the course of development. Further, relatively small alterations in receptor levels are sufficient to impact behavior,^24, 26^ indicating that naturally occurring variation in receptor levels may contribute to individual differences in behavior. Given the multiple lines of evidence implicating variation in 5-HT1A levels in mental illness, there is a clear need to identify the factors that contribute to variation in 5-HT1A expression across the lifetime to guide individualized treatment and intervention.

Studies of the transcriptional regulation of the serotonin 1a receptor gene (*HTR1A*) have identified a putatively functional G/C single-nucleotide polymorphism (SNP) located 1019 bp upstream of the translation start site, designated rs6295.^28^ The G-allele of this SNP was initially found to be overrepresented in suicide,^29^ and was also associated with a decreased responsiveness to antidepressants.^30^ As with many gene associations, replications of these results have been inconsistent, but a series of recent meta-analyses supports an association between rs6295 and mood disorders, specifically MDD and bipolar disorder.^31, 32, 33^ In addition, this SNP has also been associated with a variety of other illnesses, including panic disorder,^16^ substance abuse,^16^ premenstrual dysphoric disorder,^34, 35^ eating disorders^36^ and schizophrenia^16^ (reviewed in ref. 28). These findings suggest that rs6295 may impact intermediate phenotypes that are common to multiple disorders.^37^

*In vitro* studies indicate that rs6295 may functionally impact *HTR1A* transcription through the altered binding of transcription factors.^28^ Specifically, the G-allele fails to bind the transcription factor, NUDR/Deaf1. This leads to higher levels of *HTR1A* transcription in raphe-derived neurons where NUDR/Deaf1 acts as a repressor, but lower levels in forebrain-derived neurons where it acts as a transcriptional activator.^38, 39^ However, this dual activity has never been demonstrated in human tissue. In addition, rs6295 impacts the binding of the factors, Hes1 and Hes5, which are expressed predominantly during neuronal differentiation.^29, 38, 40^ As such, the impact of this polymorphism on transcription during early development may differ from its effects during adulthood.

To clarify the relationship between rs6295, gene transcription and mental illness, we undertook dual lines of research. First, we investigated the hypotheses that the rs6295G-allele may be associated with an increased risk for psychiatric illness, and that it may interact with exposure to trauma in a previously characterized cohort studied for the effects of trauma on anxiety and depression-related traits.^41, 42, 43^ In separate studies, we also tested the hypothesis that rs6295 may impact transcription in the human brain, using relative allelic expression assays to examine rs6295-associated transcriptional differences across the different brain regions and in early development compared with adulthood. Finally, we investigated the hypothesis that *HTR1A* transcription may be altered in disease states. Together, these studies further indicate that naturally occurring common variation in *HTR1A* may contribute to transcriptional differences and risk for psychiatric illness.

## Materials and methods

### rs6295, trauma exposure and psychiatric outcomes

#### Participants

This study was part of a larger study undertaken by the Grady Trauma Project investigating the genetic and trauma-related risk factors for posttraumatic stress disorder (PTSD) and depression in an urban, highly traumatized, low-income, predominantly African American population in Atlanta, Georgia.^41, 42, 43^ The participants were recruited while waiting for outpatient appointments at the primary care or obstetrical-gynecological clinics of Grady Memorial Hospital. Both phenotype data and rs6295 genotype data were available for 1367 subjects. Of these, a subset of 1233 self-identified African American individuals were included in the final analyses because of clear genetic stratification that corresponded with self-reported ethnicity (Supplementary Methods and Supplementary Figure 1). Given the high rates of non-replication of gene association studies, these individuals were randomly split into a discovery cohort (*n*=822) and a replication cohort (*n*=411) when assessing the relationship between rs6295 and psychiatric outcomes (Supplementary Methods). These subjects provided informed consent, completed a battery of self-report measures and provided a salivary sample for DNA extraction. Owing to variation in literacy across subjects, all self-report measures were obtained by interview. The study was approved by the institutional review boards for Emory University School of Medicine and Grady Memorial Hospital. Subsequent analysis was performed on de-identified data. Sociodemographic information included sex, age, self-identified race/ethnicity, education, employment status, income, marital status and disability status (Table 1). The mean (s.d.) age in the sample was 40.11 (13.4, range 18–77). Details of trauma and psychiatric assessments, genetic methods and data analysis are provided in Supplementary Materials and Methods.

### rs6295 and transcription in postmortem human tissue

#### Samples

Adult tissue samples were obtained from the Postmortem Brain Core Facility at the University of Mississippi Medical Centre and from Columbia University/New York State Psychiatric Institute (Table 2). Samples from the University of Mississippi were genotyped as outlined above with Taqman genotyping assay for rs6295. Samples from Columbia University had been previously genotyped for rs6295.^16^ The procedures used for brain tissue collection, toxicology, psychological autopsy and stereology have been published elsewhere.^14, 17, 44, 45, 46^ Fetal tissue samples were obtained from the NICHD Brain and Tissue Bank for Developmental Disorders at the University of Maryland. Initially, we genotyped 27 fetal samples from peripheral tissue using the Wizard Genomic DNA Purification Kit (Promega #A1120, Madison, WI, USA) according to the manufacturer's instructions followed by genotyping as outlined above. This yielded five individuals with usable haplotypes for allelic expression assays (below), and cortical tissue was subsequently obtained for these five samples. An overview of sample characteristics is shown in Table 3.

#### Identification of SNPs for allelic expression assays

To identify an SNP in the *HTR1A* mRNA that could reasonably serve as a proxy for rs6295, which is located upstream of the transcription start sites,^47^ we cloned and sequenced a region encompassing rs6295, rs6294, rs878567 and rs6449693 from 50 rs6295GC heterozygous individuals (13 fetal, 37 adults). The latter three SNPs are located in the *HTR1A* mRNA. We used primers 5′-GAGTAAGGCTGGACTGTTAGATG-3′ and 5′-AGCAGATTCGTGCATAAGGATGG-3′ in failsafe PCR premix D (Epicentre Technologies, Madison, WI, USA) and *Taq* polymerase using the following PCR conditions: 95 °C for 5 min, (95 °C for 15 s, 55 °C for 15 s, 72 °C for 4 min) × 35° cycles, 72 °C for 5 min, 4 °C forever. The PCR products were analyzed by Sanger sequencing using the following primers: 5′-GAGTAAGGCTGGACTGTTAGATG-3′, 5′-CGTGGCCAATTATCTTATTGGC-3′ and 5′-AGCAGATTCGTGCATAAGGATGG-3′. The presence of overlapping peaks at the SNP locations was used to confirm heterozygosity. In samples heterozygous for rs6295 and one of the other SNPs, we cloned the subsequent PCR product into the PCR-TopoII vector according to the manufacturer's instructions (Invitrogen #K4610-20, Carlsbad, CA, USA). Three clones were initially picked for each sample and sent for sequencing. In a subset of cases, sequencing results that were inconsistent with those of the PCR product itself were observed, presumably due to mispaired annealing and extension of a partially completed PCR product. In those instances, an additional five clones were picked and sent for sequencing and the majority haplotype from all clones was used to assign haplotypes for an individual.

#### RNA extraction

RNA from frozen tissue punches or slide scrapings were collected directly into lysis buffer and pulverized by hand using a disposable pestle (Fisher #NC9719656). RNA was extracted using the Norgen RNA/DNA Purification Kit (Norgen Biotek #48700, Thorold, ON, Canada) according to the manufacturer's instructions with the exception of 600 μl rather than 300 μl of lysis buffer. RNA quality and concentration were assessed with an RNA nano chip using a 2100 Bioanalyzer (Agilent Technologies, Santa Clara, CA, USA).

#### Assessment of allelic imbalance

Because rs6295 was found to be in much stronger LD with rs878567 and rs6449693 than with rs6294 in our samples, we investigated allelic imbalance in individuals who were heterozygous for both rs6295 and rs878567 (see Results). We chose to use rs878567 instead of rs6449693 because the T-allele of this SNP creates a trinucleotide string that appears as a single peak in the pyrogram, making it difficult to calculate relative allelic expression. The complementary DNA (cDNA) was generated with 250 ng RNA template and random primers using the high-capacity reverse transcription kit (Applied Biosystems, Foster City, CA, USA) according to the manufacturer's instructions. 1 μl cDNA was used as template for subsequent pyrosequencing reactions, which were performed in triplicate. The region surrounding rs6295 was amplified using primers 5′- TGGCTCAGACTTTGCCTGTAT-3′ and biotin 5′-AACATCCACCGCAAAGATTTAG-3′ in a 25 μl reaction containing 200 nm primers, Taq polymerase (New England Biolabs, Ipswitch, MA, USA) and premix F (Epicentre Biotechnologies) using the following reaction conditions: 95 °C for 1 min; 35 cycles: 72 °C for 15 s, 53 °C for 15 s, 72 °C for 30 s; 72 °C for 7 min. Pyrosequencing was performed on a PyroMark Q24 pyrosequencer using sequencing primer 5′-CATCAGTTTTGATCCCAG-3′ with the following order of nucleotide addition: GTCATGCT. Genomic DNA from rs878567 and cDNA from rs878567TT and CC homozygotes were used to validate the pyrosequencing assay (representative pyrograms are shown in Supplementary Figure 3). Relative percent of T and C were quantified using PyroMark Q24 2.0.4 software (Qiagen, Hilden, Germany). Allelic imbalance was determined by a one-sample *t*-test relative to an expected value of 0.5, representing equal expression from the C- and T-allele. In addition, group differences were assessed using a two- tailed Student's *t*-test.

## Results

### rs6295, trauma exposure and psychiatric outcomes

**Figure 1:**
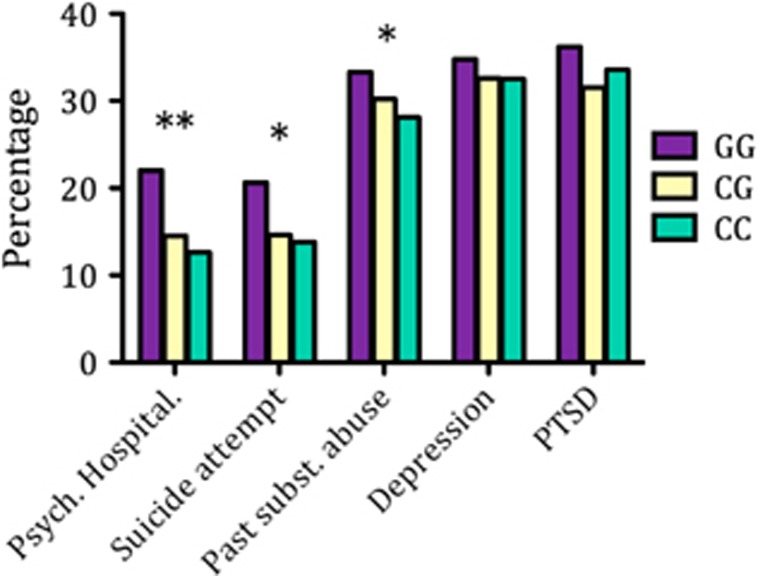
Prevalence of psychiatric outcomes for rs6295 genotypes. *P<0.05; GG versus C-allele carriers in discovery cohort. Psych., psychiatric; PTSD, posttraumatic stress disorder; subst., substance.

Our initial investigation in our discovery cohort revealed a significant association between rs6295GG genotype and psychiatric hospitalization, prior suicide attempts and previous substance abuse after controlling for sex, age, age^2^ and current disability status (Figure 1 and Table 4). In particular, rs6295GG individuals had an increased likelihood of past drug or alcohol abuse (odds ratio (OR)=1.506, *P*=0.02, confidence interval (CI)=1.068–2.122), prior hospitalization for psychiatric reasons (OR=1.571, *P*=0.025, CI=1.058–2.332) and increased likelihood of attempting suicide (OR=1.555, *P*=0.031, CI=1.040–2.323). We subsequently tested for these associations in our discovery cohort, where we confirmed a significant relationship between rs6295 and psychiatric hospitalization with a greater odds ratio than in our discovery cohort (OR=2.397, *P*=0.003, CI=1.355–4.240). In addition, although the reduced power in our replication sample failed to produce significance at the *P*<0.05 level, the odds ratios for the effect of rs6295GG and substance abuse (OR=1.126, *P*=0.645) and suicide attempts (OR=1.506, *P*=0.161) fell within the 95% CI of these associations in the discovery cohort, indicating that effect size did not differ between cohorts. The strongest and most consistent association existed between rs6295GG and psychiatric hospitalization, which may potentially reflect a pan-diagnostic impact on mental illness. We did not observe a significant association between rs6295 and current PTSD or depression.

Given the association of rs6295 with more than one psychiatric outcome, we undertook pairwise comparisons of the entire sample to determine the extent to which we observed comorbidity in our sample. Chi-squared analyses showed that all phenotypes were significantly associated with each other (*P*<0.001). Odds ratio calculations (Table 5) show that the strongest associations exist between PTSD and depression and between psychiatric hospitalization and suicide attempts. This reflects the fact that suicide attempts are the most common reason for psychiatric hospitalization.

**Figure 2:**
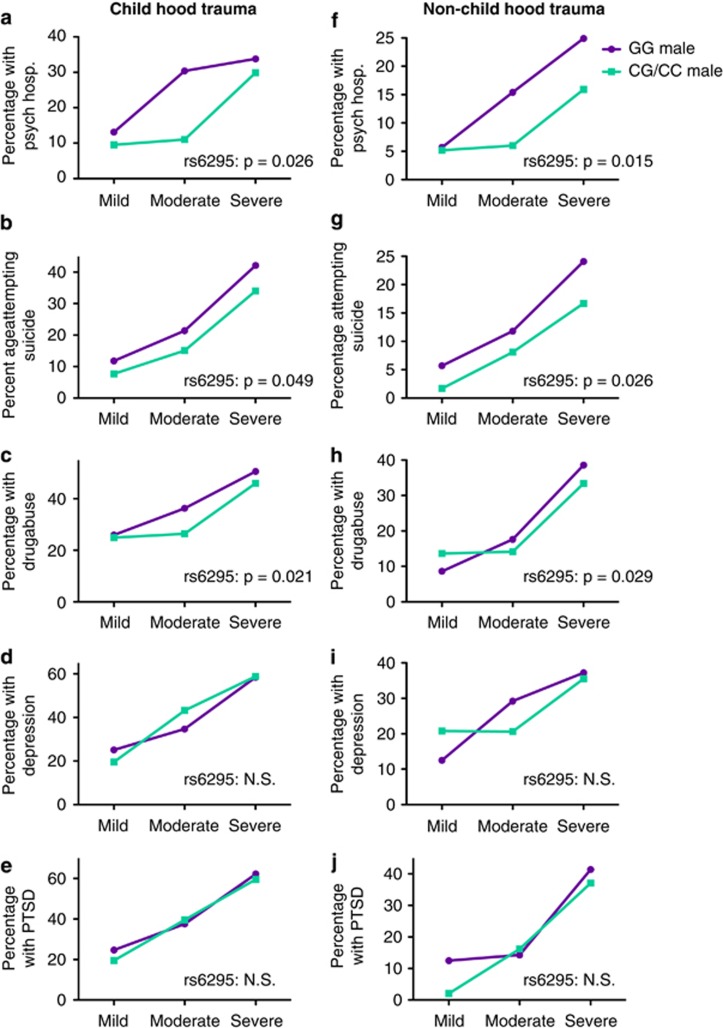
Rates of psychiatric outcomes by genotype with increasing trauma severity. Childhood trauma (**a**–**e**) and non-childhood trauma (**f**–**j**) were binned into severity categories based on the number and types of trauma encountered. Because we identified a genotype × gender interaction for PTSD, males (solid lines) and females (dashed lines) are shown separately. Significant *P*-values from the discovery cohort for main effects of rs6295G-allele are indicated on the appropriate graph. NS, not significant; PTSD, posttraumatic stress disorder.

Having investigated the potential main effects of rs6295 on psychiatric outcomes, we were also interested in identifying potential interactions with childhood or non-childhood trauma that might moderate genetic effects. We assessed potential interactions between genotype, gender and exposure to trauma for our five outcome measures. Genotype, sex, age, age^2^ and current disability status were retained in all models with successive model selection for various interactions. No significant associations were observed, but both childhood and non-childhood trauma exposure were significantly associated with increased incidence in all five psychiatric outcomes that were assessed in this study (Table 6 and Table 7 and Figure 2).

### rs6295 and transcription

To identify SNPs in the *HTR1A* gene that existed in linkage disequilibrium with rs6295 in our sample, we cloned alleles from 50 rs6295GC individuals and determined haplotypes for rs6295, rs6294, rs6449693 and rs878567, the latter three of which exist in the *HTR1A* mRNA. Table 8 shows our observed haplotype frequencies.

**Figure 3:**
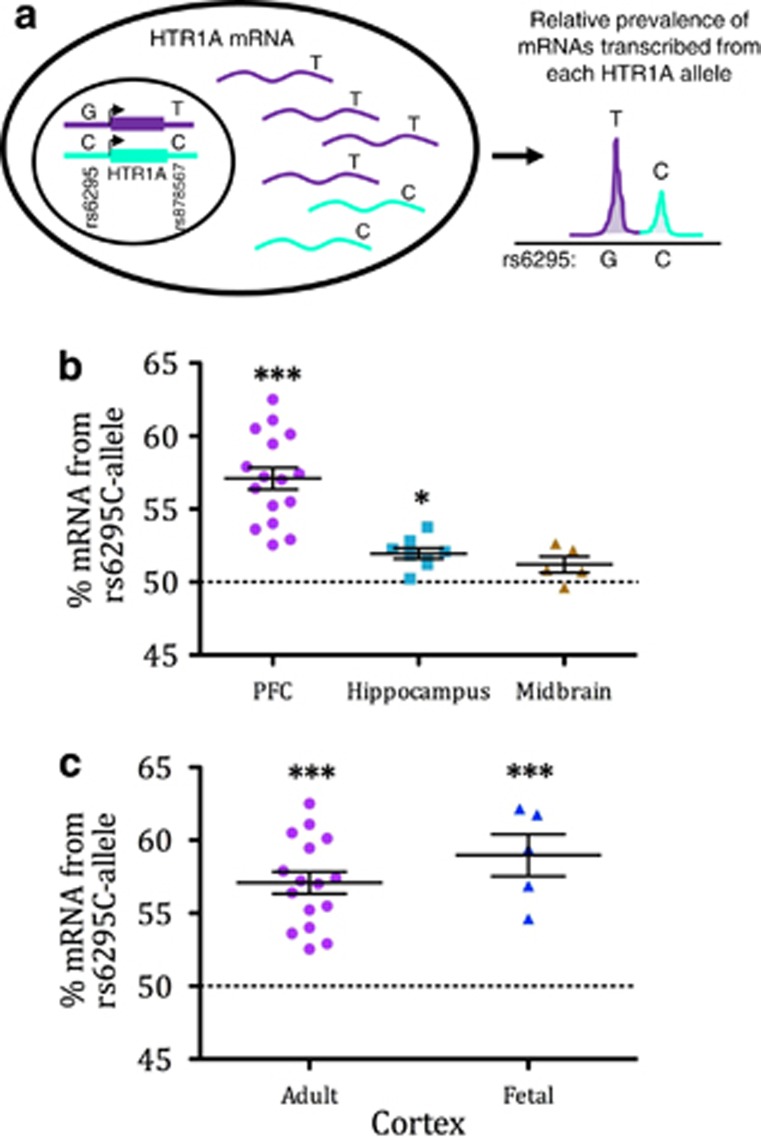
Relative allelic expression from the rs6295C-allele. (**a**) Diagrammatic representation of relative allelic expression of human *HTR1A* gene. (**b**) More mRNA was produced from the C-allele than the G-allele of rs6295 in the adult prefrontal cortex (PFC), but this was not observed in the midbrain. (**c**) Biased transcription from the C-allele was also observed in the fetal cortex. ***P<0.001, *P<0.05.

We observed strongest linkage disequilibrium between rs6295 and rs878567/rs6449693. Thus, we used rs878567 to determine the relative amount of mRNA produced from the G- versus C-allele of rs6295 in individuals heterozygous for both SNPs. Using this technique, we found more mRNA is produced from the C-allele as compared with the G-allele of rs6295 in the prefrontal cortex (PFC, Broadman areas 8 and 9) of control individuals (*t* (15)=6.092, *P*<0.001) and in the hippocampus (*t* (7)=5.194, *P*=0.003), although the effect size was much smaller in the latter (Figure 3). In addition, allelic transcription was not significantly biased in the midbrain (*t* (4)=2.881, *P*=0.135), indicating region-specific associations between allelic transcriptional differences and rs6295.

Further, because different transcription factors are thought to interact with rs6295 during neuronal differentiation, as compared with adulthood, we also investigated relative allelic expression in cortex from gestational week 18–19 fetus. Hippocampus and midbrain were not available at these early time points. As in the adult PFC, we found that more mRNA was produced from the C-allele of rs6295 (*t* (4)=6.24, *P*=0.003).

**Figure 4:**
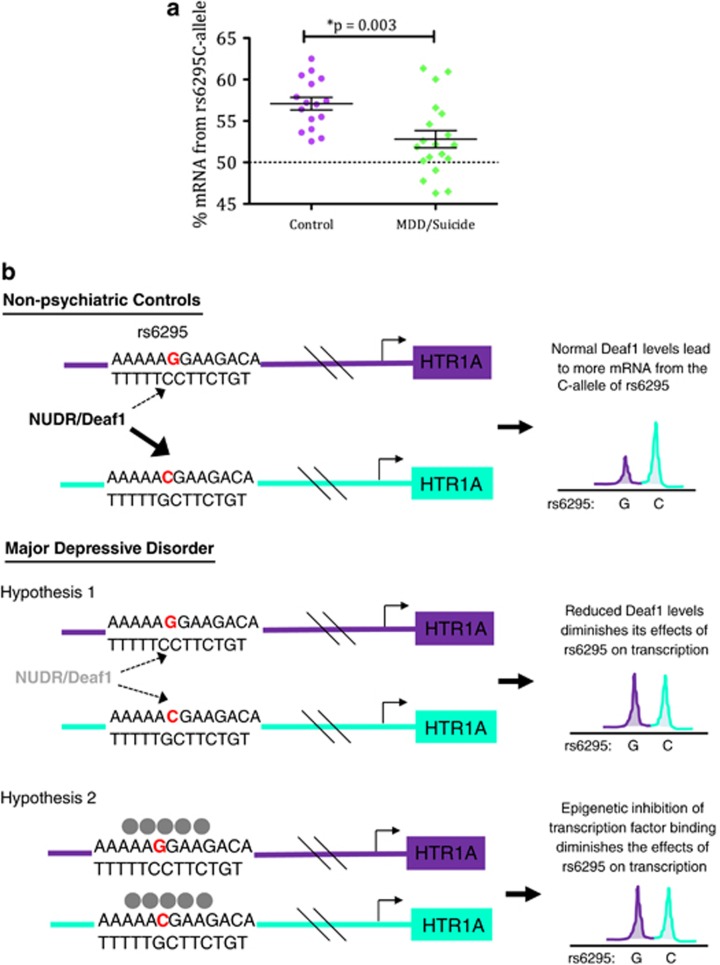
Relative allelic expression from the rs6295C-allele in the PFC of controls and MDD/suicide patients. (**a**) Relatively less *HTR1A* mRNA is produced from the C-allele of MDD patients compared with nonpsychiatric controls. (**b**) Two models that may explain the reduced penetrance of rs6295 in MDD. Previous work suggests that Deaf1 levels are impacted by stress, and decreased Deaf1 would be expected to preferentially decrease expression from the C-allele. Alternatively, epigenetic changes that inhibit access of transcription factors to the rs6295 locus would also neutralize the effects of this SNP. MDD, major depressive disorder; PFC, prefrontal cortex; SNP, single-nucleotide polymorphism.

Finally, as differences in 5-HT1A levels have been observed in the individuals who died by suicide, we also assessed relative allelic expression in a subset of individuals who had MDD (with or without suicide; Figure 4). Because MDD with (*n*=14) and without (*n*=5) suicide did not differ (*t* (17)=−1.980, *P*=0.064), these were subsequently grouped together. Individuals with MDD were significantly different in their allelic transcription profile (% mRNA produced from C-allele) as compared with controls (*t* (33)=3.235, *P*=0.003).

## Discussion

Our data provide biological support for the functional contribution of rs6295 to mental illness. In accordance with previous findings, we found that rs6295GG individuals had an increased incidence of substance and alcohol abuse, and an increased likelihood of both psychiatric hospitalization and attempting suicide. As evidence that rs6295 may directly impact these traits or their underlying intermediate phenotypes, we found that the G-allele was associated with lower levels of transcription in the PFC both during early gestation and in adulthood. These effects on transcription, which were more penetrant in nonpsychiatric controls than in patients with MDD, may thus serve as a risk factor for mental illness.

In addition to the main effects of rs6295 on psychiatric outcomes, we also explored the role of trauma. We found that exposure to either childhood or non-childhood trauma substantially increased the risk for all the psychiatric outcomes examined by us. However, we did not observe a significant interaction between rs6295 genotype and trauma exposure. It is possible that this may be owing to insufficient power, and it would be interesting to investigate potential interactions in a larger data set.

Together, these data confirm previous findings that the rs6295G allele is associated with mental illness. Furthermore, the strong association of rs6295GG genotype with increased rates of hospitalization may indicate that this genotype impacts intermediate phenotypes that span diagnostic categories and contribute to hospitalization, such as psychosis, impulsivity, aggression or disease severity. The 5-HT1A agonism is a feature of multiple pharmacotherapies, including aripiprizole, vilazadone and buspirone for schizophrenia, depression and anxiety disorders, respectively, suggesting complex and diverse behavioral effects resulting from the activation of this receptor.^19, 20, 48, 49^

We were initially surprised that we did not observe increased rates of depression in G-allele carriers, especially as GG individuals had higher likelihood of suicide attempts. However, both PTSD and depression symptoms were assessed only for 2 weeks before the data collection, while the other outcomes were retrospectively assessed across the lifespan. We do not have access to cumulative rates of PTSD or depression, and lifetime incidence of these disorders may show a stronger and/or clearer association with rs6295.

We also sought to establish a potential biological mechanism that could explain the association between rs6295 and mental illness. Because previous *in vitro* work indicated that rs6295G allele impairs the binding of a variety of transcription factors,^38, 50^ we examined the relationship between rs6295 and *HTR1A* transcription. There is often inherently high inter-individual variability in human samples that can result both from differences in life history and from differences in tissue handling, such as postmortem interval (time from death to freezing of brain tissue). As a control for this, we examined relative allelic expression in rs6295GC heterozygotes, a technique that compares the relative amount of mRNA produced from each allele in the same individual. This intra-individual comparison also has the advantage of assessing gene expression in the same transcription factor environment such that any differences in transcription are attributable to *cis*- regulatory sequence variants, such as rs6295.

Using this technique, our data indicate that rs6295, or a nearby linked variant, contributes to differences in *HTR1A* mRNA levels *in vivo*. Specifically, we found that more mRNA was produced from the C-allele of rs6295 as compared with the G-allele in the PFC of nonpsychiatric controls, but that this relative difference in expression did not occur in the raphe, although the sample sizes for hippocampus and raphe should be considered exploratory. These data support previous *in vitro* studies that found that NUDR/Deaf1 acts as a transcriptional activator in forebrain-derived cell lines.^38^ In this case, its failure to bind the G-allele would lead to decreased *HTR1A* mRNA from this allele. Although we did not observe an inversion of the C:G expression ratio in the raphe, this may be owing to insufficient spatial or cellular resolution, or it may also reflect our relatively limited sample size.

Our data further indicate that the relative increase in cortical expression from the C-allele is also present in early gestational development in our small sample set, indicating that rs6295 may impact *HTR1A* mRNA both during neuronal differentiation and later in life. *HTR1A* expression levels are high during this gestational period, and it has been hypothesized that 5-HT1A may be involved in the neurotrophic actions of serotonin.^51^ Because Hes1, Hes5, Hes6 and Deaf1/NUDR may all putatively have an impact on expression,^38, 40^ we used publicly available data sets to examine the relative levels of these genes during gestational week 18–19 (refs 52, 53; Supplementary Figure 4). At gestational week 17–18, Hes1 levels are falling, Hes5 levels are low, and Hes6 and Deaf1/NUDR levels are high. Thus, Hes1 and Deaf1 are most likely to impact *HTR1A* expression at this time. However, the extremely high levels of Hes 6 may counteract the repressive effects of Hes1.^40^ Thus, the increased expression from the C-allele at this time point may reflect a Deaf1/NUDR-dependent effect.

Finally, we also compared the C:G expression ratio in depressed suicide cases as compared with controls. Previous studies have identified an overall difference in 5-HT1A levels in depression.^13, 15, 18, 54, 55^ We focused on the PFC, as this is where we observed the largest effect for rs6295. Overall, we observed significantly less bias in the C:G relationship in MDD patients as compared with controls. Because the effects of rs6295 on transcription are stronger in nonpsychiatric controls, this SNP likely contributes to disease vulnerability rather than representing a disease state *per se*.

The blunted C:G transcriptional ratio in MDD patients suggests a change in transcription factor and/or epigenetic milieu at this locus in the disease state. Reduced Deaf1 protein has previously been observed in the PFC of depressed subjects and was correlated with reduced 5-HT1A protein levels.^56^ A reduction in Deaf1 would be expected to preferentially reduce the expression of the C-allele to neutralize allelic imbalance (Figure 4b). Alternatively, differences in epigenetic factors, such as methylation, at the locus could inhibit transcriptional access to the rs6295-containing region and thereby neutralize the effect of this polymorphism. Numerous studies have shown stress-induced differences in neural 5-HT1A levels,^57, 58, 59, 60, 61, 62, 63^ and a preliminary study in primary blood leukocytes found a correlation between *HTR1A* promoter DNA methylation levels and HTR1A mRNA levels,^64^ suggesting that epigenetic mechanisms may contribute to *HTR1A* transcriptional regulation. It is also plausible that altered Deaf1 regulation may interact with epigenetic changes in a complex, stress-dependent manner. For instance, chronic mild stress in male rats led to reduced PFC levels of 5-HT1A receptor as wellas of Deaf1,^65^ whereas more severe chronic restraint stress increased PFC *HTR1A* mRNA and 5-HT1A protein levels with no effect on Deaf1.^66^ Furthermore, different types of stress affected Deaf1 and 5-HT1A receptor expression differently and gender-specifically.^65^

There are a number of ways in which rs6295-associated alterations in 5-HT1A expression could potentially affect PFC neural function and behavior. 5-HT1A is an inhibitory receptor that is expressed both on pyramidal cells and on inhibitory interneurons in the PFC. It remains unclear whether one or both of these populations are sensitive to the putative effects of rs6295, and it has previously been suggested that the relative expression of 5-HT1A receptors on these two types of neurons may have important implications for overall effects of serotonin on limbic circuitry.^67^ In addition, there are two primary means by which the PFC has been implicated in serotonin-related behavior. First, the PFC is thought to have an important role in top-down control of limbic circuits.^68^ Alterations in 5-HT1A may impact such top-down control. In addition, the PFC provides important feedback that modulates the firing of the serotonergic raphe neurons.^69^ This feedback is sensitive to 5-HT1A activation. Thus, increased levels of 5-HT1A in the PFC may indirectly affect serotonergic modulation of other brain regions through this feedback mechanism. For instance, previous genetic imaging studies have found that rs6295 is associated with differences in amygdala activation, which the authors propose may be owing to differences in serotonin release in the amygdala.^70, 71^ Additional studies are needed to determine both the cell-type-specific effects of rs6295 in the PFC and clarify these potential circuit-level effects. Existing mouse models that enable both region-specific and temporally specific suppression of 5-HT1A levels, may be particularly well suited for this task.^26, 27, 72^

Our studies have focused on the effects of rs6295 on transcription with the assumption that this is the most direct method for investigating potential functional effects of this SNP. However, other factors beyond transcription likely also have a role in generating functional diversity within the 5-HT1A system. For instance, functional desensitization of these receptors, such as that induced by chronic antidepressant administration, may also have an important role in generating individual variation in the 5-HT1A system.^73^ Ultimately, a more thorough understanding of the diverse mechanisms impacting 5-HT1A levels and function will be important for understanding the contribution of this system to disease.

These findings provide an important advance in our understanding of a putatively functional promoter polymorphism in a psychiatrically relevant gene by linking genetic association data with an *in vivo* mechanism. The strongest association we observed was between rs6295 and psychiatric hospitalization. This may suggest that rs6295 impacts one or more intermediate phenotypes relevant to a variety of illnesses. In addition, we have provided important evidence that this SNP may have a functional role in disease risk by modulating transcription of *HTR1A* in a region-specific manner in humans. These studies provide an important framework for future studies of the complex mechanisms underlying *HTR1A*-mediated disease risk and add to the growing literature highlighting the complex mechanisms underlying individual sensitivity to stress and mental illness.

## Figures and Tables

**Table 1:** Sample demographics for epidemiological cohort

	*Participants*	
	N	*%*
*Education*		
Less than 12th grade	294	23.9
High school or equivalent	550	44.7
More than high school	385	31.2

*Employment*		
Currently employed	348	28.3
Unemployed	881	71.7

*Current disability status*		
Current receiving support	249	20.3
Not receiving support	975	79.7

*Household income (monthly)*		
$0–249	324	27.2
$250–499	120	10.1
$500–999	336	28.2
$1000 or more	412	25.6

**Table 2:** Distribution of genotypes

	*rs6295 genotype*		
	*GG*	*CG*	*CC*
	*Number (%)*	*Number (%)*	*Number (%)*
Male	145 (30.2)	243 (50.6)	92 (19.2)
Female	251 (33.3)	363 (48.2)	139 (18.5)
Total	396 (32.1)	606 (49.1)	231 (18.7)

Genotypes are in Hardy–Weinberg equilibrium for all the groups. *P*>0.05.

**Table 3:** Postmortem sample demographics

	*Control*	*MDD/suicide*
Age (year)	52.7 (±18.7)	54.9 (±17.6)
Gender	13 F, 26 M	8 F, 13 M
PMI (h)	19.3 (±9.8)	20.0 (±6.06)
Brain pH	6.5 (±0.33)	6.6 (±0.30)
RIN	7.2 (±1.5)	6.9 (±1.7)

Abbreviations: MDD, major depressive disorder; PMI, postmortem interval; RIN, RNA integrity number.

**Table 4:** Effects of rs6295 on psychiatric outcomes

	*Psychiatric hospitalization*		*Suicide attempt*		*Past substance abuse*		*Depression PTSD*		*PTSD*	
	*OR*	P	*OR*	P	*OR*	P	*OR*	P	*OR*	P
*Discovery sample*										
rs6295GG	**1.571**	**0.025**	**1.555**	**0.031**	**1.506**	**0.020**	1.234	0.203	1.282	0.128
Male sex	0.693	0.075	**0.307**	**<0.001**	**1.813**	**<0.001**	0.771	0.116	0.913	0.576
Age	**1.228**	**<0.001**	**1.237**	**<0.001**	**1.427**	**<0.001**	**1.128**	**0.004**	**1.103**	**0.019**
Age^2^	**0.998**	**0.001**	**0.998**	**0.001**	**0.996**	**<0.001**	**0.998**	**0.004**	**0.999**	**0.017**
Disability	**2.39**	**<0.001**	**2.297**	**<0.001**	1.113	0.589	1.087	0.685	1.287	0.206

*Test sample*										
rs6295GG	**2.397**	**0.003**	1.506	0.161	1.126	0.645				
Male sex	0.864	0.623	**0.542**	**0.046**	**1.609**	**0.048**				
Age	**1.428**	**<0.001**	**1.348**	**<0.001**	**1.487**	**<0.001**				
Age^2^	**0.996**	**<0.001**	**0.996**	**<0.001**	**0.996**	**<0.001**				
Disability	**3.515**	**<0.001**	**2.107**	**0.028**	1.521	0.142				

Abbreviation: PTSD, posttraumatic stress disorder.

Odds ratios (OR) and *P*-values (*P*) for *P*<0.05 are shown in bold.

**Table 5:** Pairwise relationships between psychiatric outcomes

	*Psychiatric hospitalization*	*Suicide attempt*	*Past substance abuse*	*Depression*
*Odds ratio*				
Suicide attempt	13.563			
Substance abuse	4.222	3.995		
Depression	2.642	3.546	1.852	
PTSD	2.798	2.797	2.057	8.696

Abbreviation: PTSD, posttraumatic stress disorder.

All pairwise comparisons are significant (*P*<0.001). Odds ratios shown above.

**Table 6:** Logistic regression modeling of rs6295 and childhood trauma

*Childhood trauma*	*Psychiatric hospitalization*		*Suicide attempt*		*Past substance abuse*		*Depression*		*PTSD*	
	*OR*	P	*OR*	P	*OR*	P	*OR*	P	*OR*	P
*Discovery sample*										
rs6295GG	**1.586**	**0.026**	**1.531**	**0.049**	**1.515**	**0.021**	1.179	0.338	1.265	0.175
Male sex	0.722	0.123	**0.34**	**<0.001**	**2.091**	**<0.001**	0.855	0.364	1.073	0.684
Age	**1.219**	**0.001**	**1.2**	**0.002**	**1.428**	**<0.001**	**1.093**	**0.044**	1.065	0.157
Age^2^	**0.998**	**0.003**	**0.998**	**0.007**	**0.996**	**<0.001**	0.999	0.053	0.999	0.168
Disability	**2.32**	**<0.001**	**2.186**	**0.001**	1.127	0.565	0.999	0.998	1.207	0.378
Moderate trauma^a^	1.569	0.073	**1.870**	**0.019**	**1.568**	**0.036**	**2.585**	**<0.001**	**2.589**	**<0.001**
Severe trauma^a^	**2.432**	**<0.001**	**4.900**	**<0.001**	**2.865**	**<0.001**	**3.596**	**<0.001**	**5.086**	**<0.001**

*Test sample*										
rs6295GG	**2.526**	**0.003**	1.507	0.184	1.241	0.419				
Male sex	1.196	0.585	0.752	0.378	**1.999**	**0.008**				
Age	**1.281**	**0.005**	**1.243**	**0.009**	**1.416**	**<0.001**				
Age^2^	**0.997**	**0.006**	**0.997**	**0.010**	**0.996**	**<0.001**				
Disability	**3.185**	**0.001**	1.893	0.074	1.507	0.167				
Moderate trauma^a^	2.019	0.071	1.879	0.086	1.126	0.698				
Severe trauma^a^	**7.338**	**<0.001**	**5.586**	**<0.001**	**3.446**	**<0.001**				

Abbreviation: PTSD, posttraumatic stress disorder.

a Relative to mild trauma.

Odds ratios (OR) and *P*-values (*P*) for *P*<0.05 are shown in bold.

**Table 7:** Logistic regression modeling of rs6295 and non-childhood trauma

*Non-childhood trauma*	*Psychiatric hospitalization*		*Suicide attempt*		*Past substance abuse*		*Depression*		*PTSD*	
	*OR*	P	*OR*	P	*OR*	P	*OR*	P	*OR*	P
*Discovery sample*										
rs6295GG	**1.655**	**0.015**	**1.591**	**0.026**	**1.484**	**0.029**	1.209	0.258	1.328	0.095
Male sex	**0.615**	**0.022**	**0.300**	**<0.001**	**1.703**	**0.002**	0.742	0.078	0.851	0.336
Age	**1.201**	**0.002**	**1.229**	**0.001**	**1.437**	**<0.001**	**1.109**	**0.018**	1.077	0.094
Age^2^	**0.998**	**0.005**	**0.998**	**0.001**	**0.996**	**<0.001**	**0.999**	**0.015**	0.999	0.072
Disability	**2.454**	**<0.001**	**2.178**	**0.001**	1.136	0.534	0.998	0.993	1.178	0.429
Moderate trauma^a^	1.964	0.332	4.601	0.053	1.361	0.510	1.238	0.586	**1.862**	**<0.001**
Severe trauma^a^	**4.246**	**0.018**	**7.502**	**0.006**	**2.485**	**0.020**	**2.016**	**0.035**	**7.276**	**<0.001**

*Test sample*										
rs6295GG	**2.231**	**0.007**	1.436	0.230	1.163	0.574				
Male sex	0.830	0.540	**0.506**	**0.029**	1.620	0.056				
Age	**1.386**	**<0.001**	**1.307**	**0.001**	**1.434**	**<0.001**				
Age^2^	**0.996**	**<0.001**	**0.997**	**0.002**	**0.996**	**<0.001**				
Disability	**3.163**	**0.001**	1.946	0.064	1.396	0.274				
Moderate trauma^a^	1.578	0.613	1.055	0.966	1.177	0.862				
Severe trauma^a^	2.254	0.291	5.799	0.090	**5.311**	**0.031**				

Abbreviation: PTSD, posttraumatic stress disorder.

a Relative to no trauma.

Odds ratios (OR) and *P*-values (*P*) for *P*<0.05 are shown in bold.

**Table 8:** Distribution of haplotypes in rs6295GC samples

*rs6295*	*rs6294*	*rs6449693*	*rs878567*	*Frequency*
C	G	T	C	50%
G	A	T	C	9%
G	G	T	C	3%
G	G	C	T	38%
