# Supplementary material for: The functional serotonin 1a receptor promoter polymorphism, rs6295, is associated with psychiatric illness and differences in transcription
Source: Transl Psychiatry. 2016 Mar 1;6(3):e746–. doi: 10.1038/tp.2015.226 (PMC4872437; doi:10.1038/tp.2015.226)
Supplement: Supplementary Information [file tp2015226x1.pdf]

Supplementary Materials and Methods.

Assessment of population stratification

In order to control for population substructure within our sample population (all ethnicities), we calculated principal components (PC) for 1172 individuals for whom whole genome SNP information was available (1). The Grady Trauma Project performed genome-wide SNP genotyping of over 5000 individuals using the HumanOmniExpress and Omni1-Quad BeadChip (Illumina Inc). The HumanOmniExpress interrogates 730,525 individual SNPs per sample, whereas the Omni1-Quad BeadChip interrogates 1,011,219 individual SNPs. We performed basic quality control measures in PLINK(2). We removed individuals with greater than 4% missing data. We further identified and removed related individuals to estimate the proportion of identity by descent (IBD) for each pair of individuals. Among pairs of individuals with an IBD proportion > 0.12 (indicating cousins or a closer relation), we preferentially removed PTSD controls over cases. We also removed SNPs with less than a 96% call rate, a frequency of less than 0.01, and Hardy-Weinberg equilibrium (HWE) p-value < 10^{-6} in PTSD controls. We pruned the autosomal SNPs in windows of 50 base pairs, removing one SNP from each pair of SNPs with r^2>0.05 to obtain a set of roughly independent SNPs (N= 63,187). We then performed principal-component analysis (PCA). Plotting PC 1 versus PC 2 revealed clear clusters that largely corresponded with self-reported ethnicity (Suppl fig 1). Thus, to avoid potential confounding effects of population stratification when examining genetic associations, we limited our analysis to individuals who self-identified as African-American.

Assessment of trauma and psychiatric outcomes

Childhood trauma was assessed retrospectively using the Childhood Trauma Questionaire (CTQ), a psychometrically-validated 28-item inventory (3, 4). Scores were calculated for

physical, emotional, and sexual abuse encountered before the age of 18. We classified participants into mild, moderate, and severe trauma categories using previously reported categorizations as follows. Participants were first classified in two groups, none to mild range, and moderate to severe range for each type of abuse as outlined above, based on Bernstein and Fink's score ranges for none, mild, moderate, and severe levels of abuse (4). Participants were subsequently divided into three categories based on the number of types of abuse (emotional, physical, or sexual) they had experienced in the moderate to severe range. Participants with no abuse in the moderate and severe range were classified as mild (n = 685, 56.8%), those with one type of abuse in the moderate and severe range were classified as moderate (n = 278, 23.0%), and those with two or more types of abuse in this range were classified as severe (n= 244, 20.2%). Trauma category was used as a categorical covariate in analyses.

Non-childhood abuse trauma exposure was assessed using the Traumatic Events Inventory (TEI), which is a 14-item screen for lifetime exposure to different categories of trauma (5, 6). These include natural disaster, serious accident or injury, sudden life threatening illness, military combat, being attacked with a weapon, witnessing a family member or friend being attacked with or without a weapon, witnessing the murder or suicide of a friend or family member, or forced sexual contact. For each traumatic event, the TEI assesses experiencing and witnessing of events separately. The childhood abuse items in this inventory were excluded to avoid overlap with information collected with the CTQ. To categorically measure non-child abuse measures with the TEI, we binned this variable into three groups, those who experienced 0, 1, or 2 or more types of non-child abuse trauma. Based on these categorizations, 7.9% (n = 94) participants reported no trauma, 12.9% (n = 153) experienced 1 type of non-child abuse trauma, and 79.2% (n = 943) experienced 2 or more types of non-child abuse trauma.

Post-traumatic stress disorder was assessed using the PTSD symptom scale (PSS). The PSS is a psychometrically validated 17-item self-report scale assessing PTSD symptomology over the two weeks prior to rating (6, 7). A categorical variable that serves as a proxy for PTSD was created based on DSM-IV A-E criterion responses to the PSS questionnaire (A, presence of trauma; B, presence of at least 1 intrusive symptom; C, presence of at least 3 avoidance, numbing symptoms; D, presence of at least 2 hyperarousal symptoms; and E, present for at least 1 month)(8, 9).

Depressed mood during the 2 week period prior to the interview was assessed using the 21-item Beck Depression Inventory (BDI). This is a well-validated and commonly used continuous measure of the level of depressive symptoms(10). Each of the items measures the presence and severity of depressive symptoms, which are rated on a scale from 0 to 3. A categorical definition of MDD was determined by a cutoff score of 15 on the BDI. In order to validate the use of BDI score as a proxy for depression, a subset of 545 individuals underwent the Structured Clinical Interview for DSM-IV Axis 1 Disorders (SCID-1)(11). In this subset, the BDI total scores for those with current MDD ($n = 88$, mean (SD) = 26.9 (13.3)) were significantly different from the BDI total scores for those without a current MDD diagnosis ($n = 397$, mean (SD) = 12.8 (10.9)) ($t(483) = -10.5$; $p < 0.001$).

Previous psychiatric hospitalizations, suicide attempts, and past drug or alcohol abuse were all self-reported, with individual yes-no items asked after the demographics.

Genetic methods

DNA was extracted as previously described(8, 12). rs6295 was genotyped using a Taqman assay (Life Technologies #4351379, Carlsbad, CA). Polymerase chain reactions were performed in 5 ul reaction volumes in 384-well plates and contained 5 ng of DNA following the standard protocol provided with the kit. 1412 samples were genotyped. Genotypes were found to be in Hardy Weinberg equilibrium as assessed using a Chi Squared test (Table 2)(13). The distribution of genotypes did not differ between males and females (n = 1233, $X^2(2) = 2.08$, p = 0.354).

Data analysis

Recent work has suggested that many genetic risk factors may be shared across psychiatric diagnostic categories(14), and as such, we examined the relationship between rs6295 and five categorical psychiatric outcome measures: current PTSD, current depression, past drug or alcohol abuse, history of psychiatric hospitalization, and previous suicide attempts. Fifty-seven samples that lacked information for two or more of these categories were excluded from the analysis. Subsequent analysis of genetic stratification indicated genetic clustering that corresponded with self-reported ethnicity (Suppl Fig 1), so we focused on a subset of 1233 individuals who self-identified as African American or Black to reduce background genetic variation. Subsequently, 822 individuals were randomly assigned to a discovery cohort where we examined all potential relationships between rs6295, psychiatric outcomes, and trauma exposure; the remaining 411 individuals were then assigned to a confirmation cohort in which only significant associations from the discovery cohort were interrogated. In instances of significant associations, individual pairwise contrasts revealed that GG allele carriers were different from both CG and CC carriers, which did not differ from each other. Thus, we adopted a dominant rather than additive genetic model, and CC and CG individuals were grouped

together in subsequent analyses. We used a logistic regression analysis to control for sex, age, age^2, and current disability status. Both age and age^2 were included because a quadratic model was a better fit for disease incidence across the life span. Current disability status was included to control for potential biological factors that could impact health and were not attributable to rs6295. rs6295 was not associated with current disability status ($X^2(1) = 0.215$, $p = 0.643$).

In order to investigate potential interactions between childhood (up to age 18) and non-childhood (age 18 and older) trauma exposure and rs6295 genotype, we performed bivariate logistic regressions to determine the main effects of rs6295, sex, age, age^2, current disability status, and either childhood or non-childhood trauma, as well as all potential two and three way interactions between TEI or CTQ, sex, and rs6295 for the five outcomes listed above. Non-significant three-way and then two-way interactions were subsequently removed from the model in a stepwise hierarchical fashion.

The final models were subjected to two sensitivity analyses. First, we included additional covariates for education level, employment, relationship status, and monthly income in the final models. Inclusion of these covariates did not affect significant effects of rs6295. Second, we calculated PCs for self-identified African American/Black subjects in order to assess potential population stratification within this subgroup (n = 1068). Plotting PC1 versus PC2 and PC3 versus PC4 revealed single clusters with a few outliers (Supp fig 2). Accordingly, we omitted subjects that were >5 standard deviations from a 10% trimmed mean for any the first four PCs. This excluded 21 individuals (16 based on PC1, 3 based on PC2, and 2 based on PC4). We then repeated all final models on this genetic subset, including PC1 – 4 and chip type as covariates in the logistic regression to further control for genetic variation. Exclusion of the genetic outliers

and incorporation of the PCs in the models did not alter the effect sizes for our associations (Supp Tables 1 and 2).

We also calculated the Pearson correlation coefficient for CTQ and TEI scores ($r = 0.337$, $p < 0.001$) and examined the pairwise associations between psychiatric outcomes using Chi squared tests (Table 5). All statistical analyses were performed on SPSS version 22.

Supplementary table 1. Effects of rs6295 on psychiatric outcomes with genetic PCs included

	Psychiatric hospitalization		Suicide attempt		Past substance abuse		Depression		PTSD	
	OR	p	OR	p	OR	p	OR	p	OR	p
rs6295GG	1.654	0.024	1.582	.040	1.449	.050	1.195	.304	1.231	.227
male sex	0.658	0.068	0.294	.000	1.859	.001	.721	.061	.861	.383
age	1.229	0.001	1.303	.000	1.416	.000	1.110	.018	1.090	.047
age^2	0.998	0.003	0.997	.000	.996	.000	.999	.018	.999	.044
disability	2.586	<0.001	2.366	.001	1.148	.523	.995	.982	1.235	.320
PC1	0.020	0.223	12.646	.493	.005	.063	2.429	.741	3.719	.627
PC2	6.170	0.592	26.116	.351	.050	.289	2.999	.677	4.298	.580
PC3	2.132	0.835	19.258	.425	3.235	.691	1.148	.960	12.053	.362
PC4	0.007	0.154	0.278	.720	.143	.501	3.310	.657	2.483	.734
Gene chip	1.547	0.075	1.239	.402	1.351	.154	.870	.491	1.135	.525

Supplementary Table 2. Logistic regression modeling of rs6295 X trauma interactions with genetic PCs included

**CHILDHOOD TRAUMA**	**Psychiatric hospitalization**		**Suicide attempt**		**Past substance abuse**		**Depression**		**PTSD**	
	OR	p	OR	p	OR	p	OR	p	OR	p
rs6295GG	1.590	0.041	1.542	0.063	1.475	.046	1.162	0.404	1.238	0.241
male sex	0.700	0.126	0.315	<0.001	2.082	<0.001	0.793	0.200	1.002	0.992
age	1.202	0.005	1.269	<0.001	1.400	<0.001	1.075	0.113	1.053	0.256
age^2	0.998	0.013	0.997	0.001	.997	<0.001	0.999	0.137	0.999	0.292
disability	2.581	<0.001	2.293	0.002	1.131	0.583	0.875	0.558	1.099	0.677
Moderate trauma[1]	1.763	0.039	1.830	0.035	1.637	0.036	2.505	<0.001	2.506	<0.001
Severe trauma[1]	2.300	0.002	4.006	<0.001	3.272	<0.001	3.406	<0.001	5.154	<0.001
PC1	0.021	0.236	17.251	0.448	.005	0.064	2.762	0.715	4.628	0.592
PC2	1.646	0.886	6.284	0.614	.008	0.102	1.171	0.954	0.826	0.946
PC3	2.521	0.805	18.692	0.453	3.513	0.681	1.270	0.934	8.945	0.453
PC4	0.003	0.098	0.150	0.605	.044	0.295	1.950	0.810	0.956	0.987
Gene chip	1.617	0.054	1.325	0.289	1.390	0.134	0.840	0.407	1.087	0.695

[1]relative to mild trauma

**NON-CHILDHOOD TRAUMA**	**Psychiatric hospitalization**		**Suicide attempt**		**Past substance abuse**		**Depression**		**PTSD**	
	OR	p	OR	p	OR	p	OR	p	OR	p
rs6295GG	1.767	0.012	1.622	0.034	1.431	0.063	1.164	0.390	1.277	0.172
male sex	0.622	0.041	0.282	<0.001	1.696	0.004	0.679	0.031	0.759	0.198
age	1.193	0.007	1.306	<0.001	1.410	<0.001	1.089	0.062	1.067	0.159
age^2	0.998	0.015	0.997	<0.001	0.996	<0.001	0.999	0.053	0.999	0.129
disability	2.613	0.000	2.284	0.001	1.108	0.642	0.896	0.628	1.125	0.593
Moderate Trauma[1]	1.030	0.968	3.539	0.119	1.010	0.984	1.275	0.567	2.523	0.168
Severe Trauma[1]	3.223	0.058	5.971	0.016	2.164	0.053	2.391	0.014	9.491	<0.001
PC1	0.107	0.533	7.914	0.587	0.024	0.215	5.522	0.550	12.705	0.394
PC2	10.809	0.506	20.667	0.400	0.019	0.177	3.690	0.632	5.101	0.558
PC3	1.416	0.926	16.854	0.456	2.219	0.792	1.484	0.888	14.765	0.343
PC4	0.003	0.108	0.320	0.753	0.102	0.438	3.499	0.649	1.780	0.836
Gene chip	1.551	0.081	1.247	0.400	1.326	0.192	0.813	0.322	1.097	0.657

[1]relative to no trauma

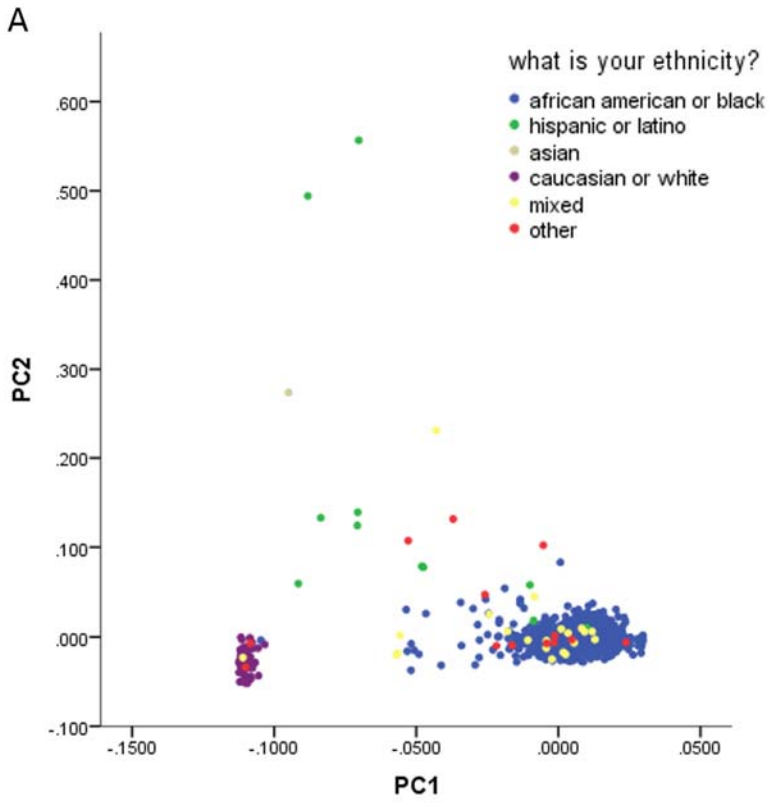

Supplementary Figure 1. Self-reported ethnicity mapped onto PC1 versus PC2. Genetic clustering largely corresponds with self-reported ethnicity.

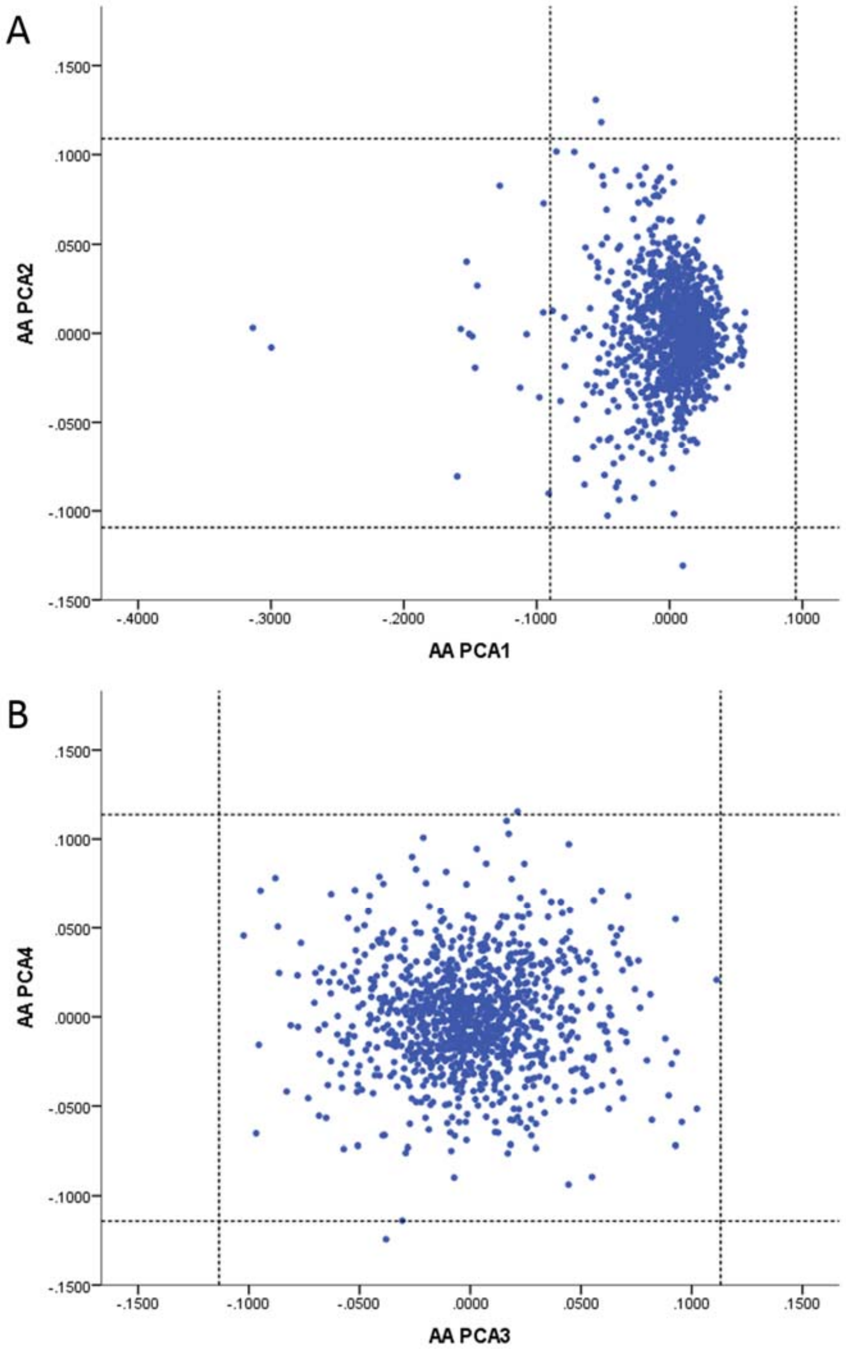

Supplementary Figure 2. Plots of genetic PC1 versus PC2 (A) and PC3 versus PC4 (B) for individuals identifying as African American/Black. Dotted lines denote 5 standard deviations from the trimmed. Subjects outside of these boundaries were excluded from the sensitivity analysis.

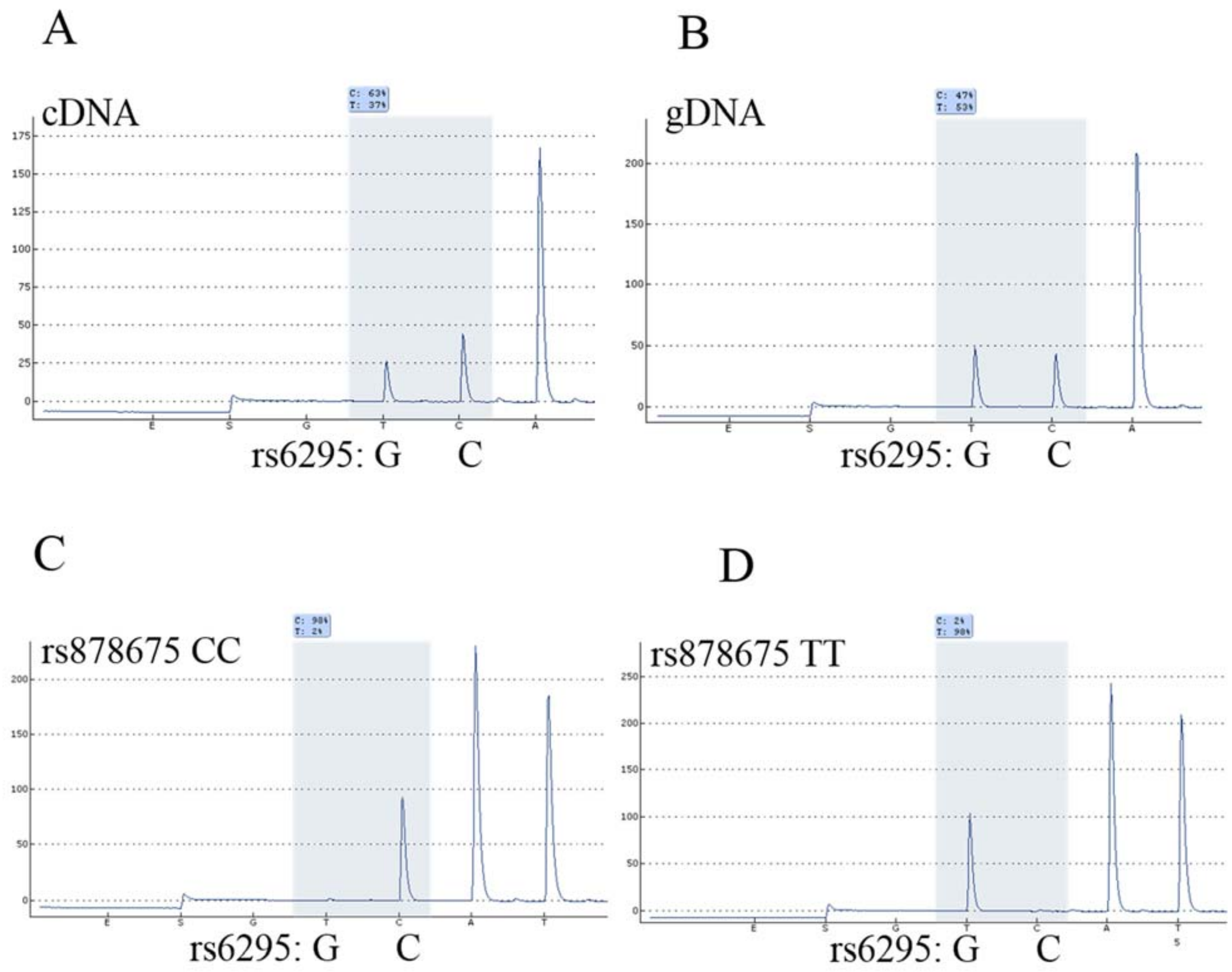

Supplementary Figure 3. Pyrosequencing provides a sensitive platform for detecting allelic expression differences in HTR1A mRNA. (A) Example pyrograms demonstrate differences in mRNA abundance for the C versus T alleles of rs878567 (as a proxy for rs6295). (B) gDNA controls show a ~50/50 ratio for T and C with a slight skew in the opposite direction of what is observed in cDNA. (C and D) No peaks are present for the opposite allele in homozygotes.

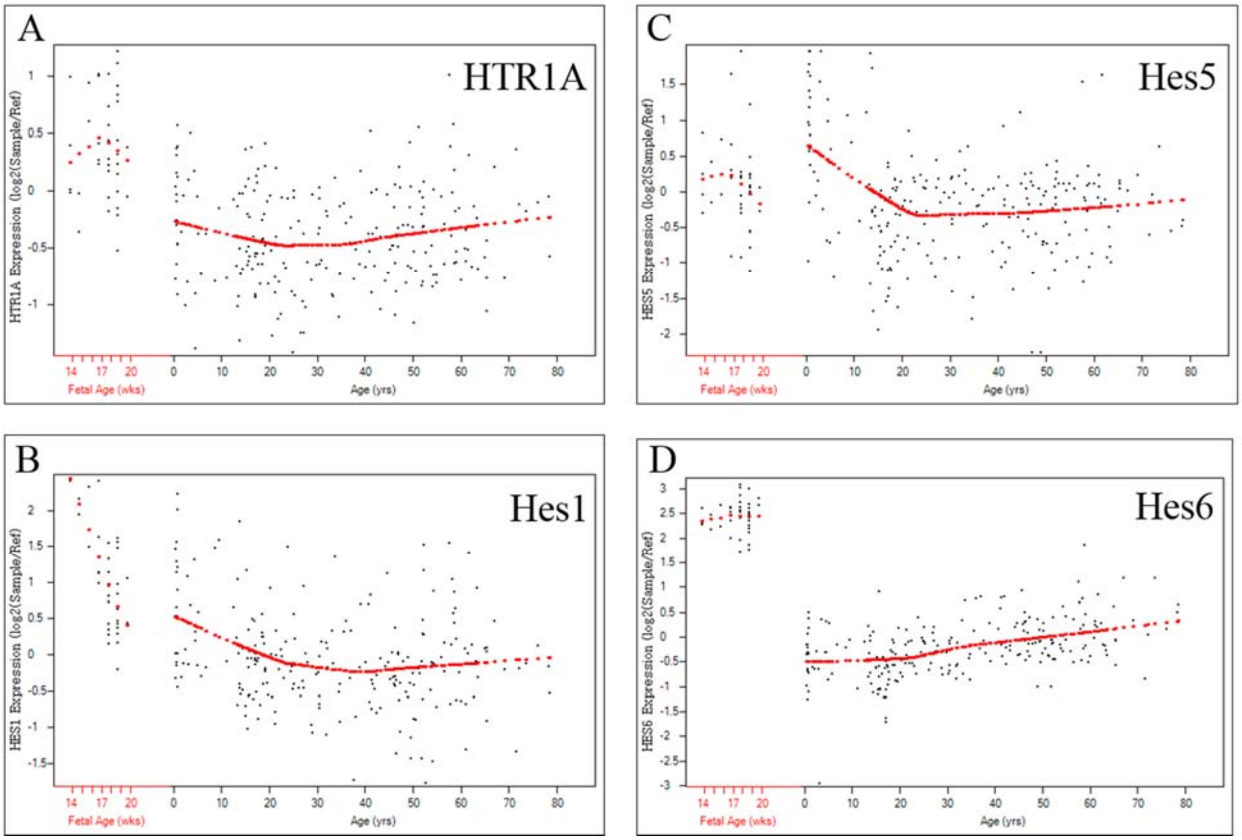

Supplementary Figure 4. Lifecourse expression of HTR1A, Hes1, Hes5, and Hes6 in the prefrontal cortex derived from Braincloud database(15). HTR1A (A) is highly expressed in week 18 – 19 fetal cortex. Hes1 expression (B) decreases from prenatal weeks 14 to 20, while Hes5 (C) exhibits relatively lower expression during these time points and higher levels at birth. Hes6 (D) levels are consistently high from prenatal weeks 14 to 20. Although Deaf1 levels were not available in this dataset, data from the Allen Brain Institute suggest that Deaf1 is also expressed during development and confirms findings for Hes1, Hes5, and Hes6 (16).

References

1. Price AL, Patterson NJ, Plenge RM, Weinblatt ME, Shadick NA, Reich D (2006): Principal components analysis corrects for stratification in genome-wide association studies. *Nat Genet*. 38:904-909.

2. Purcell S, Neale B, Todd-Brown K, Thomas L, Ferreira MA, Bender D, et al. (2007): PLINK: a tool set for whole-genome association and population-based linkage analyses. *Am J Hum Genet*. 81:559-575.

3. Bernstein DP, Stein JA, Newcomb MD, Walker E, Pogge D, Ahluvalia T, et al. (2003): Development and validation of a brief screening version of the Childhood Trauma Questionnaire. *Child Abuse Negl*. 27:169-190.

4. Bernstein DP, Fink L (1998): *Childhood Trauma Questionnaire Manual*. San Antonio, TX: Psychological Corp.

5. Gillespie CF, Bradley B, Mercer K, Smith AK, Conneely K, Gapen M, et al. (2009): Trauma exposure and stress-related disorders in inner city primary care patients. *Gen Hosp Psychiatry*. 31:505-514.

6. Schwartz AC, Bradley RL, Sexton M, Sherry A, Ressler KJ (2005): Posttraumatic stress disorder among African Americans in an inner city mental health clinic. *Psychiatr Serv*. 56:212-215.

7. Foa EB, Tolin DF (2000): Comparison of the PTSD Symptom Scale-Interview Version and the Clinician-Administered PTSD scale. *Journal of traumatic stress*. 13:181-191.

8. Ressler KJ, Mercer KB, Bradley B, Jovanovic T, Mahan A, Kerley K, et al. (2011): Post-traumatic stress disorder is associated with PACAP and the PAC1 receptor. *Nature*. 470:492-497.

9. Binder EB, Bradley RG, Liu W, Epstein MP, Deveau TC, Mercer KB, et al. (2008): Association of FKBP5 polymorphisms and childhood abuse with risk of posttraumatic stress disorder symptoms in adults. *JAMA : the journal of the American Medical Association*. 299:1291-1305.

10. Beck AT, Ward CH, Mendelson M, Mock J, Erbaugh J (1961): An inventory for measuring depression. *Arch Gen Psychiatry*. 4:561-571.

11. First M, Spitzer R, Gibbon M, Williams J (1996): *Structured Clinical Interview for DSM-IV Axis 1 Disorders, Research version.* New York: Biometrics Research Dept, New York State Psychiartic Institute.

12. Bradley RG, Binder EB, Epstein MP, Tang Y, Nair HP, Liu W, et al. (2008): Influence of child abuse on adult depression: moderation by the corticotropin-releasing hormone receptor gene. *Arch Gen Psychiatry*. 65:190-200.

13. Rodriguez S, Gaunt TR, Day IN (2009): Hardy-Weinberg equilibrium testing of biological ascertainment for Mendelian randomization studies. *Am J Epidemiol*. 169:505-514.

14. Smoller JW, Craddock N, Kendler K, Lee PH, Neale BM, Nurnberger JI, et al. (2013): Identification of risk loci with shared effects on five major psychiatric disorders: a genome-wide analysis. *Lancet*. 381:1371-1379.

15. Colantuoni C, Lipska BK, Ye T, Hyde TM, Tao R, Leek JT, et al. (2011): Temporal dynamics and genetic control of transcription in the human prefrontal cortex. *Nature*. 478:519-523.

16. ABI (2014): Brainspan: Atlas of the developing human brain.
